# A novel electrocoagulation electrode configuration for the removal of total organic carbon from primary treated municipal wastewater

**DOI:** 10.1007/s11356-020-08678-4

**Published:** 2020-04-16

**Authors:** Alaa H. Hawari, Afnan M. Alkhatib, MhdAmmar Hafiz, Probir Das

**Affiliations:** 1 Department of Civil and Architectural Engineering, College of Engineering, Qatar University, 2713, Doha, Qatar; 2 Algal Technologies Program, Center for Sustainable Development, College of Arts and Sciences, Qatar University, 2713, Doha, Qatar

**Keywords:** Electrocoagulation, Dielectrophoresis, Primary treated wastewater, Electrode configuration, Total organic carbon (TOC)

## Abstract

In this paper, the removal of total organic carbon (TOC) from a primary treated municipal wastewater using a new electrode configuration in electrocoagulation was evaluated. The used electrode configuration induces a dielectrophoretic (DEP) force by using an asymmetrical aluminum electrode with an alternating current power supply. The impact of applied current, electrolysis time, and interelectrode distance on the removal efficiency of TOC were evaluated. The experimental results showed that the maximum removal efficiency of TOC was obtained at 30 min electrolysis time, 600 mA applied current, and 0.5 cm interelectrode distance. Under these operating conditions, the TOC removal was 87.7% compared to 80.5% using symmetrical aluminum electrodes with no DEP effect. The energy consumption at the selected operating conditions was 3.92 kWh/m^3^. The experimental results were comparable with the simulation results done by COMSOL Multiphysics software.

## Introduction

Electrocoagulation is being used to treat wastewaters generated from different industries such as heavy oil refinery, textile industry, dairy industry, and distillery (Krishna et al. 2010; Kushwaha et al. 2010; Mallesh 2018; Wei et al. 2010; Zodi et al. 2010). The main reason for its wide range of application is its easy operation, low investment and maintenance cost, low sludge volume, and low space requirements (Mallesh 2018). The high zeta potential of suspended particles in wastewaters prevents the agglomeration of these particles (López-Maldonado et al. 2014). Adding coagulants to the wastewater reduces the zeta potential of such particles allowing van der Waals force to become the more dominant force which promotes agglomeration of particles and allows formation of flocs, hence removal by gravity settling (Duan and Gregory 2003). In electrocoagulation (EC), coagulants are produced from a sacrificial anode made from iron or aluminum (Zhao et al. 2014). When electricity is passed, Fe^2+^ or Al^3+^ is produced from the anode and these ions produce metal hydroxides in wastewater, which act as coagulants (Xu and Zhu 2004).

Several studies have been investigated the efficiency of electrocoagulation in treating various wastewater types at different operating conditions. Aoudj et al. (2010) targeted the treatment of textile industrial wastewater by EC with a DC power system using two aluminum symmetrical plates. Applying a current density of 1.875 mA/cm^2^, 98% of dye was removed after 60 min of electrolysis time (Aoudj et al. 2010). Bener et al. (2019) used EC for the treatment of real textile water with a DC power supply system using four aluminum and iron sheets. Applying a current density of 25 mA/cm^2^, 42.5, 18.6, 83.5, 64.7, and 90.3–94.9% of TOC, COD, turbidity, TSS, and color removal efficiencies were obtained (Bener et al. 2019). Kobya and Demirbas (2015) used EC for the treatment of can manufacturing wastewater using four aluminum sheets in a monopolar parallel mode. Applying a current density of 2 mA/cm^2^, removal efficiencies of 99.41, 99.38, 99.8, 72, and 37% of Al, Zr, phosphate, COD, and TOC were achieved after 40 min of electrolysis time (Kobya and Demirbas 2015). Saleem et al. (2011) studied the treatment of municipal wastewater by EC with a DC power system using symmetrical sheets. Applying a current density of 24.7 mA/cm^2^, 77.2% of COD was removed after 30 min of electrolysis time (Saleem et al. 2011). Kamaraj et al. (2013) investigated the impacts of an alternating and direct current on the effectiveness of EC to treat copper from wastewater using two magnesium symmetrical sheets. Applying a current density of 0.025 A/dm^2^, 97.8%, and 97.2% of copper was removed using alternating current and direct current, respectively (Kamaraj et al. 2013). All previous studies used symmetrical electrodes in electrocoagulation process. According to the findings from our previous studies, the use of asymmetrical electrodes in electrocoagulation can improve the removal of pollutants from wastewater and can reduce the corrosion of electrodes. Alkhatib et al. (2020) evaluated the use of two asymmetrical cylindrical aluminum electrodes in electrocoagulation for the removal of total phosphorus (TP) and chemical oxygen demand (COD) from treated municipal wastewater (Alkhatib et al. 2020). The removal of TP and COD increased by 24% and 18%, respectively, using the asymmetrical electrodes compared to the conventional EC process. In addition, the electrode corrosion was reduced by 87% when using the asymmetrical electrodes in the EC process. In addition, Hawari et al. (2020) investigated the impact of using the asymmetrical cylindrical aluminum electrodes in an EC process for the harvesting of marine microalgae (Hawari et al. 2020). It was found that the new electrodes configuration reduced the amount of aluminum content in the harvested biomass by 52% compared to the conventional EC electrodes.

In this study, a new flat sheet asymmetrical aluminum electrodes configuration is proposed for the removal of total organic carbon (TOC) from primary treated municipal wastewater. The performance of symmetrical and asymmetrical electrodes configurations with alternating current power supply was evaluated. In addition, the new electrodes configuration was simulated using COMSOL software.

## Materials and methods

### Wastewater characterization

The wastewater used in this study was collected from a municipal wastewater treatment plant in Doha. The wastewater samples were collected after the primary settling tanks. The characteristics of the collected wastewater samples along with the standard measuring methods are summarized in Table 1.

**Table 1:** Characteristics of wastewater samples collected after primary settling and the standard measurement method

Parameters	Initial value	Standard measurement method
Conductivity (mS/cm)	2.29±0.01	APHA 2510 B. conductivity
Temperature (°C)	22.2±0.1	APHA 2550 temperature
pH	7.03±0.01	APHA 4500-H+ B. electrometric method
TOC (mg/L)	121.9±0.06	APHA 5310 total organic carbon
TSS (mg/L)	80±20	APHA 2540 D. total suspended solids dried at 103–105 °C
Turbidity (NTU)	158±25	APHA 2130 B. nephelometric method

### Experimental setup

**Fig. 1:**
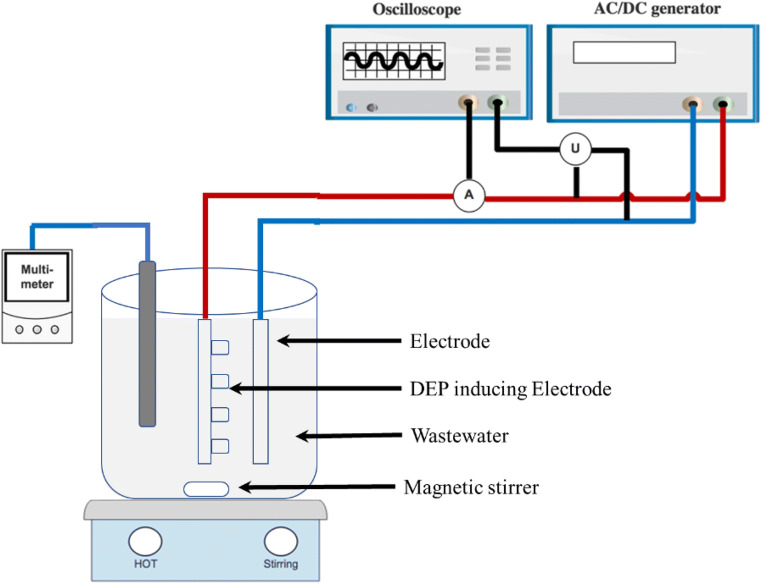
Experimental setup of the electrocoagulation reactor

**Fig 2:**
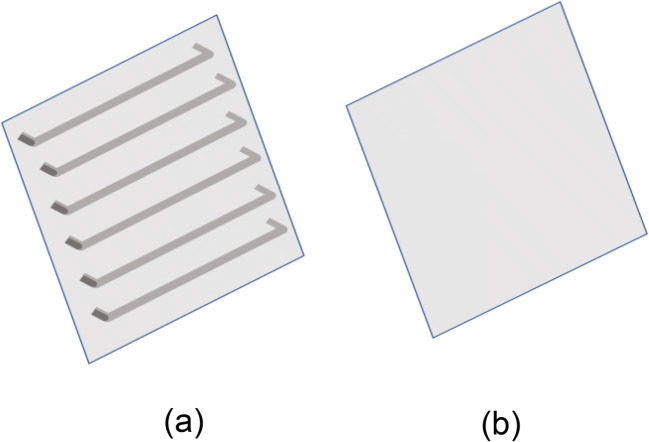
The used electrodes: **a** DEP-inducing electrode, **b** normal electrode

Figure 1 shows the used electrocoagulation reactor with a working volume of 800 mL. The solution was mixed by a magnetic stirrer at a speed of 100 rpm. Two different electrode configurations were tested. Module 1 used symmetrical electrodes with an alternating current power system. Module 2 used unsymmetrical electrodes with an alternating current power system. Module 1 will be referred to as AC. Module 2 will be referred to as AC-DEP. In the AC module, the setup consists of two aluminum symmetrical sheets with dimensions of 7 cm × 5 cm. In the AC-DEP module, one of the electrodes had parallel rods along the width of the electrode with a diameter of 2 mm (Fig. 2a), the other electrode consisted of a flat aluminum sheet (Fig. 2b). In the AC-DEP module, the dimensions of both electrodes were kept the same as the AC module. Both the AC and AC-DEP modules were connected to a VARIAC transformer providing a voltage within 0 to 250 V at a constant frequency of 50 Hz. A TEKTRONIX mixed domain oscilloscope was used to measure the voltage and current. The AC system was set to generate a sine wave.

The energy consumption in kWh/m^3^ was calculated using the following equation:

1 $$ \mathrm{Energy}\ \mathrm{consumption}=\frac{\mathrm{Voltage}\ \left(\mathrm{V}\right)\times \mathrm{current}\ \left(\mathrm{amp}\right)\times \mathrm{time}\ \left(\mathrm{h}\right)}{\mathrm{volume}\ \left({\mathrm{m}}^3\right)} $$

### Numerical methods

Dielectrophoretic (DEP) force is defined as the movement of free particles by dielectric polarization in an inhomogeneous electric field (Hawari et al. 2015). For a spherical particle, the applied dielectrophoretic force can be calculated as (Du et al. 2018; Hawari et al. 2015):

2 $$ {F}_{\mathrm{DEP}}=4\uppi {\mathrm{a}}^3{\upvarepsilon}_0{\upvarepsilon}_{\mathrm{M}}\mathrm{re}\left[\overset{\sim }{\mathrm{K}}\right]\left(E\bullet \nabla \right)E $$

where, *a* is the particle radius, ε_0_ is the permittivity of free space ($8.854\times {10}^{-12}\frac{F}{m}$), ε_*M*_is the absolute permittivity of the medium, $\mathrm{re}\left[\overset{\sim }{\mathrm{K}}\right]$ is the Clausius-Mossotti (CM) factor, and E is the field intensity ($\frac{V}{m}\Big)$, where $\mathrm{re}\left[\overset{\sim }{\mathrm{K}}\right]$ can be calculated by (Hawari et al. 2015):

3 $$ \overset{\sim }{\mathrm{K}}=\frac{\overset{\sim }{\varepsilon_p}-\overset{\sim }{\varepsilon_M}}{\overset{\sim }{\varepsilon_p}+2\overset{\sim }{\varepsilon_M}} $$

4 $$ \overset{\sim }{\upvarepsilon}=\varepsilon -\frac{\mathrm{j}\upsigma}{\upomega} $$

where, $\overset{\sim }{\varepsilon_p}$ is the complex permittivity of the particle, $\overset{\sim }{\varepsilon_M}$ is the complex permittivity of the medium, $\overset{\sim }{\varepsilon }$ is the complex permittivity, *σ* is the conductivity ($\frac{S}{m}\Big)$, *ω* is the angular frequency ($\frac{\mathrm{rad}}{s}\Big)$, *j* is the geometric gradient of the square of electric field (*E*), which can be calculated by (Hawari et al. 2015):

5 $$ j=\sqrt{-1}\cdotp \left(E\bullet \nabla \right)E=\frac{1}{2}\nabla {\left|E\right|}^2 $$

Negative DEP is expected in this study as the permittivities of the particles are higher than the permittivity of the surrounding medium (primary treated effluent) (Hawari et al. 2015).

**Fig. 3:**
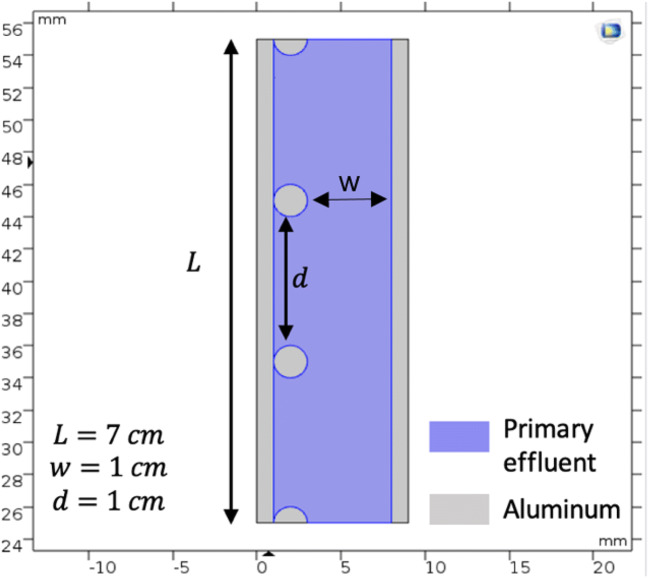
Illustration of the simulated setup with geometrical parameters

Two unsymmetrical aluminum electrodes were used in order to induce DEP force in the electrocoagulation process. The dimensions of the DEP-inducing aluminum electrode that has been used in the simulation are shown in Fig. 3. As shown in Equation (2), the square electric field is directly related with the DEP force. Therefore, the square electric field between the two electrodes surfaces was simulated using the COMSOL Multiphysics software as an indicator of the DEP force. The simulation was performed in two dimensions, assuming the width of the electrodes is going to infinity. In order to calculate the electric field exerted on the particles, the electric potential was solved at a set of boundary conditions. The medium was assumed to be the used wastewater. In order to be able to solve this problem for the applied currents, the quasi-electrostatic form was used. The root mean square (rms) of the electric field is given by the following:

6 $$ E=-\nabla \varphi $$

where *φ* is the root mean square (rms) of the electrostatic potential which can be given by Laplace’s equation (assuming that the medium is liquid only with the absence of the particles and it is homogeneous):

7 $$ {\nabla}^2\varphi =0 $$

Fixed boundary conditions were applied on the surface of the electrodes:

8 $$ {\varphi}_1={\mathrm{U}}_{\mathrm{o}} $$

9 $$ {\varphi}_2=0 $$

where *U*_0_is the rms of the oscillating potential drop. In order to ensure mesh-independent results, adaptive mesh refinement has been applied.

## Simulation results

### Impact of current on the distribution of the DEP force field

**Fig. 4:**
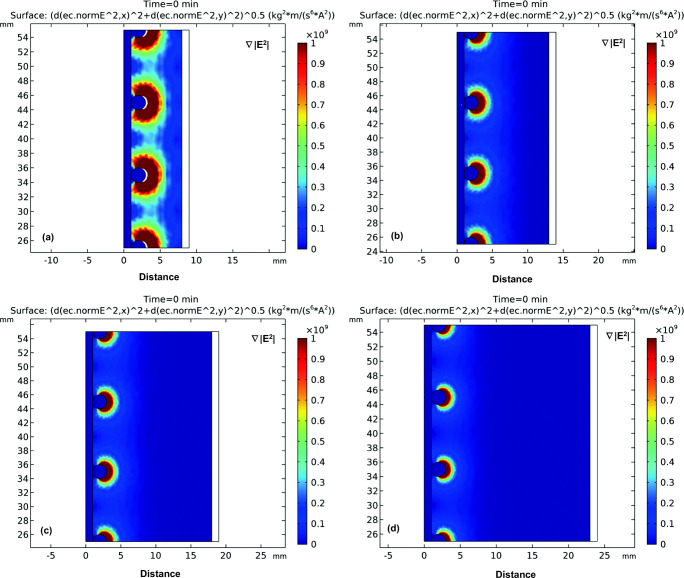
The distribution of the DEP force field defined as (∇|*E*|^2^) for the four different applied current of **a** 200 mA, **b** 400 mA, **c** 600 mA, and 800 mA; interelectrode distance: 1 cm

**Fig. 5:**
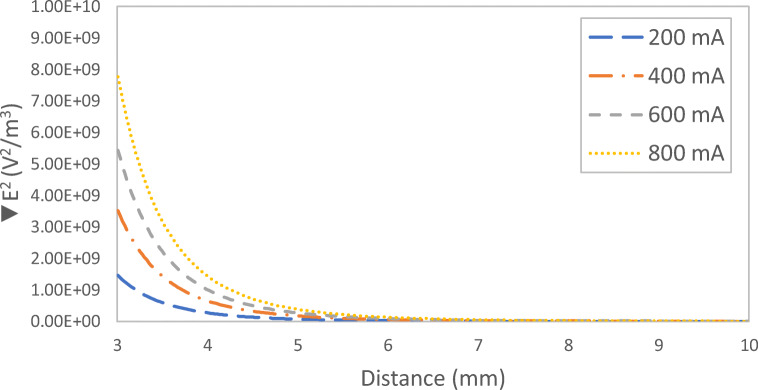
Electric field squared distribution between the two electrodes for different applied current; interelectrode distance: 1 cm

Figure 4 shows the impact of current on the distribution of the DEP force field. It can be noticed from Fig. 4 that the DEP force is being generated from the rods attached to the electrode. The DEP force induced by applying a current of 200 and 400 mA was not very significant with an interelectrode distance of 1.0 cm (Fig. 4a and b). At higher applied current of 600 and 800 mA, the induced DEP force was more significant as can be seen in Fig. 4c and d. Figure 5 shows the electric field squared distribution between the electrodes at different applied current. As can be noticed from Fig. 5 that increasing the current will cause the generated DEP force to increase(Hawari et al. 2015; Larbi et al. 2018). In addition, it can be noticed that the DEP force was almost zero at a distance of 5 mm away from the DEP inducing electrode at all applied currents.

### Impact of interelectrode distance on the distribution of the DEP force field

**Fig. 6:**
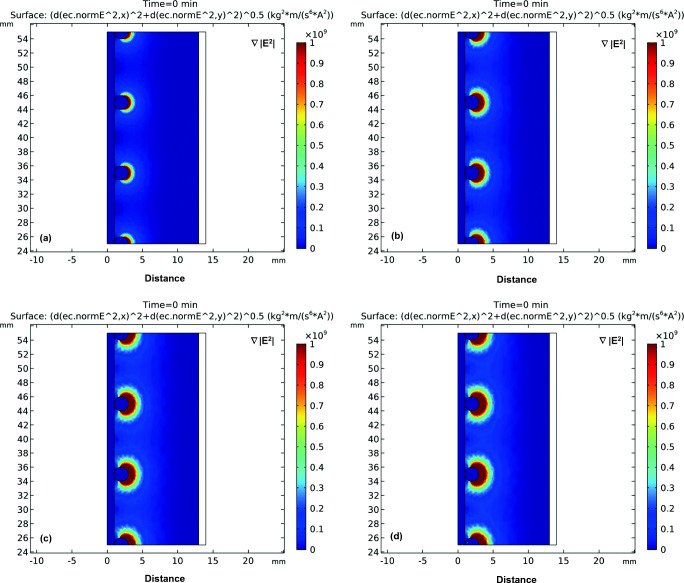
The distribution of the DEP force field defined as (∇|*E*|^2^) for four different interelectrode distance of **a** 0.5 cm,(**b** 1 cm, **c** 1.5 cm, and 2 cm; current: 600 mA

**Fig. 7:**
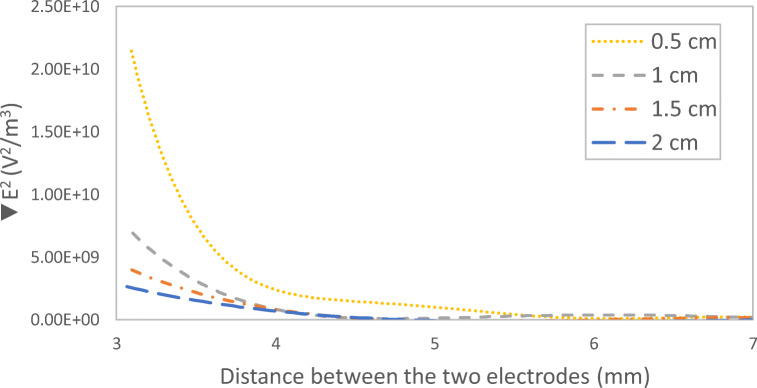
Electric field squared distribution between the two electrodes for different interelectrode distance; current: 600 mA

Figure 6 shows the impact of interelectrode distance on the distribution of the DEP force field. It can be noticed from Fig. 6 that the DEP force induced by a 0.5-cm interelectrode distance was the highest. The strength of electric field squared distribution followed a descending order of 0.5 cm, 1 cm, 1.5 cm, and 2 cm interelectrode distance as can be seen in Fig. 7. Therefore, as the distance between the two unsymmetrical electrodes increases, the generated DEP force decreases. It can be also seen from Fig. 6 that for a 1-cm, 1.5-cm, and 2-cm interelectrode distance, almost 4 mm away from the DEP-inducing electrode, the impact of the generated DEP force was minimal. However, in the 0.5-cm interelectrode distance, the impact of the DEP force extends to a 5-mm distance away from the electrode. Thus, the simulation results show that the electrode configuration with a 0.5-cm interelectrode distance is expected to have the highest DEP effect.

## Experimental results and discussion

### Impact of electrolysis time on TOC removal efficiency

**Fig. 8:**
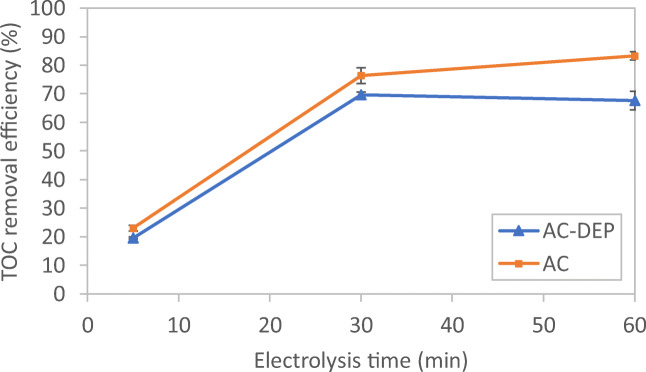
Impact of electrolysis time on TOC removal efficiency for both symmetrical and unsymmetrical electrodes configurations; current: 400 mA; interelectrode distance: 1 cm

Figure 8 shows the impact of electrolysis time on TOC removal efficiency for the AC and the AC-DEP modules at a 400-mA applied current and a 1-cm interelectrode distance. At a 5-min electrolysis time, TOC removal efficiency for the AC and the AC-DEP modules were low at 23.0% and 19.6%, respectively. The removal efficiency of TOC increased as the electrolysis times increased. It can be seen that at an electrolysis time of 60 min, the TOC removal efficiency increased to 83.3% and 67.7% for the AC and the AC-DEP modules, respectively. The TOC removal efficiency depends directly on the amount of the metal hydroxides formed from the dissolution of electrodes. As the electrolysis time increases, the formation of metal hydroxides increases leading to the increase in the TOC removal efficiency (Kobya and Delipinar 2008). It could be also seen from Fig. 8 that the AC module gave higher TOC removal efficiency than the AC-DEP module at all studied electrolysis times. This could be due to the fact that the DEP force was minimal and had no impact on the TOC removal efficiency. As shown from the simulation results, at an applied current of 400 mA and at an interelectrode distance of 1.0 cm, the DEP force was minimal. It can be also seen from Fig. 8 that TOC removal efficiency reached equilibrium almost after 30 min electrolysis time. Thus, 30 min electrolysis time was chosen as an optimum electrolysis time in the present study.

### Impact of applied current on TOC removal efficiency

**Fig. 9:**
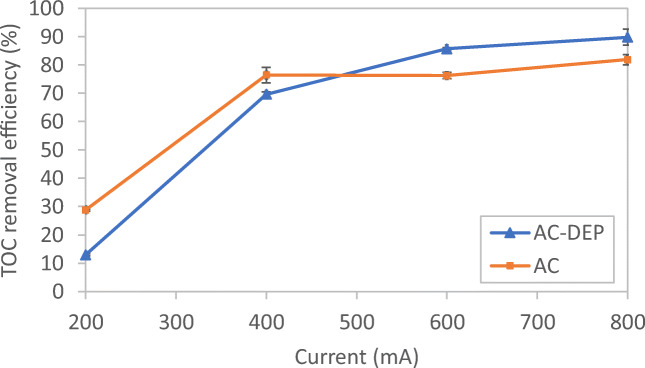
Impact of current on TOC removal efficiency for both symmetrical and unsymmetrical electrodes configurations; electrolysis time: 30 min; interelectrode distance: 1 cm

**Fig. 10:**
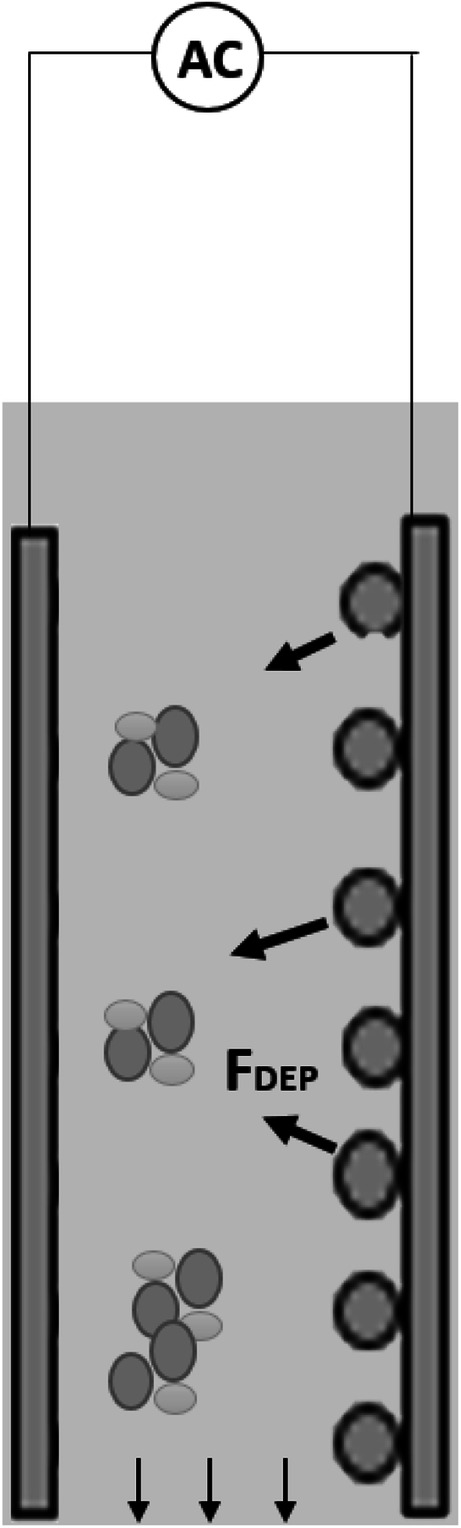
Schematic description of the enhancement of the formation of flocs using the DEP force

Figure 9 shows the impact of applied current on TOC removal efficiency at a 30-min electrolysis time and a 1-cm interelectrode distance. Generally, for both AC and AC-DEP modules as the applied current increases, the TOC removal efficiency increases. Increasing the applied current increases, the production of metal ions which generate amorphous flocs (Al(OH)3). This enhances the agglomeration of organic and inorganic matters, which leads to the increase in TOC removal efficiency (Bener et al. 2019; Kobya and Delipinar 2008). It can be also noticed from Fig. 9 that at low current of 200 and 400 mA, the AC module gave a slightly higher TOC removal efficiency than the AC-DEP module. The removal efficiency of TOC using the AC-DEP module depends on two main factors: the production of aluminum ions and the magnitude of the DEP force. When the current was below 600 mA, the production of aluminum ions and DEP force were low; therefore, the removal efficiency of TOC using the AC-DEP module was lower than the AC module. In the AC module, the removal efficiency of TOC depends only on the production of aluminum ions as no DEP force will be exerted. As shown from the simulation results, at low applied current, the generated DEP force was low (Fig. 4a and b). However, at higher currents of 600 and 800 mA, TOC removal efficiency obtained by the AC-DEP module was higher than that for the AC module. Where at an applied current of 600 mA, the TOC removal efficiency was 85.7% and 76.2 for the AC-DEP module and the AC module, respectively. At an applied current of 800 mA, the TOC removal efficiency was 89.9% and 81.8% for the AC-DEP module and the AC module, respectively. This could be due to the fact that at higher applied current, the DEP force was large enough to repel the contaminants away from the electrode surface, preventing electrode passivation and enhancing the formation of agglomerates that can be removed by gravity settling. Figure 10 illustrates the impact of DEP on particles agglomeration and settling. Since beyond the 600 mA applied current, there was no significant enhancement in the TOC removal efficiency for both modules. The 600 mA applied current was considered in studying the impact of the interelectrode distance on TOC removal efficiency.

### Impact of interelectrode distance on TOC removal efficiency

**Fig. 11:**
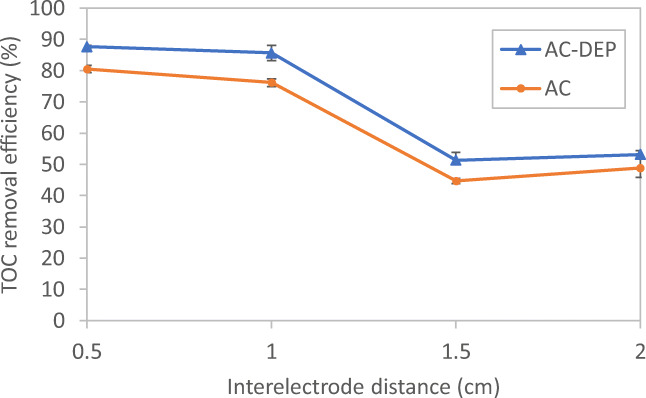
Impact of interelectrode distance on TOC removal efficiency for both symmetrical and unsymmetrical electrodes configurations; electrolysis time: 30 min; current: 600 mA

Figure 11 shows the impact of interelectrode distance on the TOC removal efficiency for four different interelectrode distances: 0.5, 1, 1.5, and 2 cm at a 30-min electrolysis time and a 600-mA applied current. It can be seen from Fig. 11 that the AC-DEP module gave higher TOC removal efficiencies when compared to the AC module at all studied interelectrode distances. It was also found that as the interelectrode distance increased, the TOC removal efficiency decreased. A maximum TOC removal efficiency of 80.5% and 87.7% was achieved at a 0.5-cm interelectrode distance for the AC module and the AC-DEP module, respectively. Further increase of the interelectrode distance lowers the TOC removal efficiency. At the maximum studied interelectrode distance of 2 cm, the TOC removal efficiency decreased to 48.9% and 53.2% for the AC module and the AC-DEP module, respectively. This result is consistent with the simulation results shown in Fig. 7. Where the highest DEP effect was found to be at an interelectrode distance of 0.5 cm. As the interelectrode distance increases, the effect of the DEP force decreases; and hence the removal efficiency of TOC decreases. In addition, as the interelectrode distance increases, the resistance in the solution between the electrodes increases which would also reduce the amount of dissociated aluminum ions. Thus, the removal efficiency decreases with increasing the interelectrode distance (Anand et al. 2014). The TOC removal efficiency of 87.7% obtained at the selected running conditions by the AC-DEP module is one of the highest TOC removal efficiencies when compared to previous studies that targeted the removal of TOC using electrocoagulation (Bektaş et al. 2004; Bener et al. 2019; Kobya and Delipinar 2008; Kobya and Demirbas 2015; Kobya et al. 2014).

Table 2 summarizes the quality of the wastewater using the most suitable running conditions. The most suitable running conditions are a AC-DEP module, a 1-cm interelectrode distance, and a 30-min electrolysis time.

**Table 2:** Characteristics of the wastewater samples before and after treatment using AC-DEP module at 600 mA applied current with an inter electrode distance of 1-cm and for 30-min electrolysis time

Parameters	After treatment
Conductivity (mS/cm)	2.23 ± 0.01
Temperature (°C)	22.5 ± 0.1
pH	7.38 ± 0.01
TOC (mg/L)	17.4 ± 0.06
TSS (mg/L)	20 ± 20
Turbidity (NTU)	14 ± 25

### Energy consumption

**Fig. 12:**
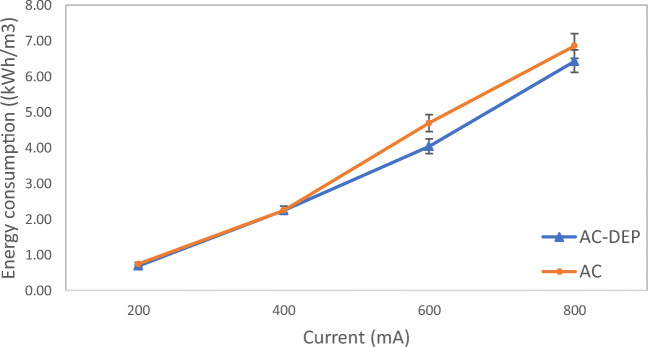
Impact of current on energy consumption for both symmetrical and unsymmetrical electrodes configurations; electrolysis time: 30 min; interelectrode distance: 1 cm

The energy consumption for the AC module and the AC-DEP module was calculated using Equation 5. Figure 12 illustrates the energy consumption using the two different electrode modules for the four applied currents 200, 400, 600, and 800 mA at a 30-min electrolysis time and a 0.5-cm interelectrode distance. It can be seen from Fig. 12 that as the applied current increased, the energy consumption increased for the AC and the AC-DEP modules. It can be also seen from Fig. 12 that at low currents of 200 and 400 mA, the AC module consumed same amount of energy as the AC-DEP module as there was minimal impact of dielectrophoresis at these low applied currents as shown from the simulation results in Figs. 4a and b. At higher applied currents of 600 and 800 mA, the AC module gave higher energy consumption than the AC-DEP module. The energy consumption at an applied current of 600 mA was 14% lower using the AC-DEP module compared to the AC module. The energy consumption at an applied current of 800 mA was 6% lower using the AC-DEP module compared to the AC module. The higher energy consumption obtained by the AC module at high applied currents of 600 and 800 mA could be due to the higher resistance in the system. It was found that the resistance in the AC system was 0.01685 Ω while in the AC-DEP system the resistance was 0.01592 Ω. The higher resistance could have caused less dissociation of metal ions and metal hydroxides into the reactor. Table 3 shows the difference in electrodes consumption between the AC-DEP module and the AC module at different applied currents. It can be seen from Table 3 that the consumption of electrodes in the AC-DEP module was always lower than the AC module. As the applied current increases, the difference in electrodes consumption between the AC-DEP module and the AC module also increases where at an applied current of 200 mA the electrode consumption in the AC-DEP module was 4.2% less than the AC module and at an applied current of 800 mA the difference increased to 27.3%.

**Table 3:** The difference in electrodes consumption between the AC-DEP module and the AC module at different applied currents

Applied current (mA)	200	400	600	800
Reduction in electrode consumption using the AC-DEP module compared to the Ac module	4.2%	9.7%	17.9%	27.3%

## Conclusion

In this paper, a dielectrophoretic (DEP) force inducing electrode configuration was proposed for the removal of total organic carbon (TOC) from primary treated municipal wastewater. The experimental results show that the effect of the generated DEP force at low currents of 200 mA and 400 mA was not sufficient to improve the TOC removal efficiency, while at higher applied currents of 600 mA and 800 mA the TOC removal efficiency was enhanced when compared to the symmetrical electrodes module (i.e., none DEP induced module). Moreover, a maximum TOC removal efficiency of 87.7% was achieved using the AC-DEP module at an applied current of 600 mA and an interelectrode distance of 0.5 cm with a 30-min electrolysis time. The energy consumption in the system was found to be 3.92 kWh/m^3^. Thus, the proposed new electrode configuration showed the ability of enhancing the electrocoagulation process compared to the conventional symmetrical electrodes configuration. In addition, it was found that consumption of electrodes in the AC-DEP module was always lower than the AC module. As the applied current increases, the difference in electrodes consumption between the AC-DEP module and the AC module also increases where at an applied current of 800 mA the electrode consumption in the AC-DEP module was 27.3% less than the AC module. DEP force inducing electrode configuration was found to economical due to lower energy consumption.

## References

[CR1] Alkhatib AM, Hawari AH, Hafiz MA, Benamor A (2020). A novel cylindrical electrode configuration for inducing dielectrophoretic forces during electrocoagulation. J Water Process Eng.

[CR2] Anand MV, Srivastava VC, Singh S, Bhatnagar R, Mall ID (2014). Electrochemical treatment of alkali decrement wastewater containing terephthalic acid using iron electrodes. J Taiwan Inst Chem Eng.

[CR3] Aoudj S, Khelifa A, Drouiche N, Hecini M, Hamitouche H (2010). Electrocoagulation process applied to wastewater containing dyes from textile industry. Chem Eng Process Process Intensif.

[CR4] Bektaş N, Akbulut H, Inan H, Dimoglo A (2004). Removal of phosphate from aqueous solutions by electro-coagulation. J Hazard Mater.

[CR5] Bener S, Bulca Ö, Palas B, Tekin G, Atalay S, Ersöz G (2019). Electrocoagulation process for the treatment of real textile wastewater: effect of operative conditions on the organic carbon removal and kinetic study. Process Saf Environ Prot.

[CR6] Du F, Hawari AH, Larbi B, Ltaief A, Pesch GR, Baune M, Thöming J (2018). Fouling suppression in submerged membrane bioreactors by obstacle dielectrophoresis. J Membr Sci.

[CR7] Duan J, Gregory J (2003). Coagulation by hydrolysing metal salts. Adv Colloid Interf Sci.

[CR8] Hawari AH, Du F, Baune M, Thöming J (2015). A fouling suppression system in submerged membrane bioreactors using dielectrophoretic forces. J Environ Sci.

[CR9] Hawari AH, Alkhatib AM, Das P, Thaher M, Benamor A (2020). Effect of the induced dielectrophoretic force on harvesting of marine microalgae (Tetraselmis sp.) in electrocoagulation. J Environ Manag.

[CR10] Kamaraj R, Ganesan P, Lakshmi J, Vasudevan S (2013). Removal of copper from water by electrocoagulation process—effect of alternating current (AC) and direct current (DC). Environ Sci Pollut Res.

[CR11] Kobya M, Delipinar S (2008). Treatment of the baker’s yeast wastewater by electrocoagulation. J Hazard Mater.

[CR12] Kobya M, Demirbas E (2015). Evaluations of operating parameters on treatment of can manufacturing wastewater by electrocoagulation. J Water Process Eng.

[CR13] Kobya M, Gengec E, Sensoy M, Demirbas PE (2014) Treatment of textile dyeing wastewater by electrocoagulation using Fe and Al electrodes: optimisation of operating parameters using central composite design. Color Technol 130

[CR14] Krishna BM, Murthy UN, Manoj Kumar B, Lokesh KS (2010). Electrochemical pretreatment of distillery wastewater using aluminum electrode. J Appl Electrochem.

[CR15] Kushwaha JP, Srivastava VC, Mall ID (2010). Organics removal from dairy wastewater by electrochemical treatment and residue disposal. Sep Purif Technol.

[CR16] Larbi B, Du F, Baune M, Thöming J, Hawari AH (2018). Numerical study on the effect of insulator size and shape on fouling suppression by electrodeless dielectrophoresis in submerged membrane bioreactors. AIP Conf Proc.

[CR17] López-Maldonado EA, Oropeza M, Jurado Baizaval JL, Ochoa-Terán A (2014). Coagulation–flocculation mechanisms in wastewater treatment plants through zeta potential measurements. J Hazard Mater.

[CR18] Mallesh B (2018) A review of electrocoagulation process for wastewater treatment

[CR19] Saleem M, Bukhari A, Akram M (2011). Electrocoagulation for the treatment of wastewater for reuse in irrigation and plantation (report). Aust J Basic Appl Sci.

[CR20] Wei L, Guo S, Yan G, Chen C, Jiang X (2010). Electrochemical pretreatment of heavy oil refinery wastewater using a three-dimensional electrode reactor. Electrochim Acta.

[CR21] Xu X, Zhu X (2004). Treatment of refectory oily wastewater by electro-coagulation process. Chemosphere.

[CR22] Zhao S, Huang G, Cheng G, Wang Y, Fu H (2014). Hardness, COD and turbidity removals from produced water by electrocoagulation pretreatment prior to reverse osmosis membranes. Desalination.

[CR23] Zodi S, Potier O, Lapicque F, Leclerc J-P (2010). Treatment of the industrial wastewaters by electrocoagulation: optimization of coupled electrochemical and sedimentation processes. Desalination.

